# A Self-Attention Integrated Learning Model for Landing Gear Performance Prediction

**DOI:** 10.3390/s23136219

**Published:** 2023-07-07

**Authors:** Lin Lin, Changsheng Tong, Feng Guo, Song Fu, Yancheng Lv, Wenhui He

**Affiliations:** School of Mechatronics Engineering, Harbin Institute of Technology, Harbin 150001, China

**Keywords:** performance prediction, feature selection, data distribution, integrated learning, self-attention

## Abstract

The landing gear structure suffers from large loads during aircraft takeoff and landing, and an accurate prediction of landing gear performance is beneficial to ensure flight safety. Nevertheless, the landing gear performance prediction method based on machine learning has a strong reliance on the dataset, in which the feature dimension and data distribution will have a great impact on the prediction accuracy. To address these issues, a novel MCA-MLPSA is developed. First, an MCA (multiple correlation analysis) method is proposed to select key features. Second, a heterogeneous multilearner integration framework is proposed, which makes use of different base learners. Third, an MLPSA (multilayer perceptron with self-attention) model is proposed to adaptively capture the data distribution and adjust the weights of each base learner. Finally, the excellent prediction performance of the proposed MCA-MLPSA is validated by a series of experiments on the landing gear data.

## 1. Introduction

**Figure 1:**
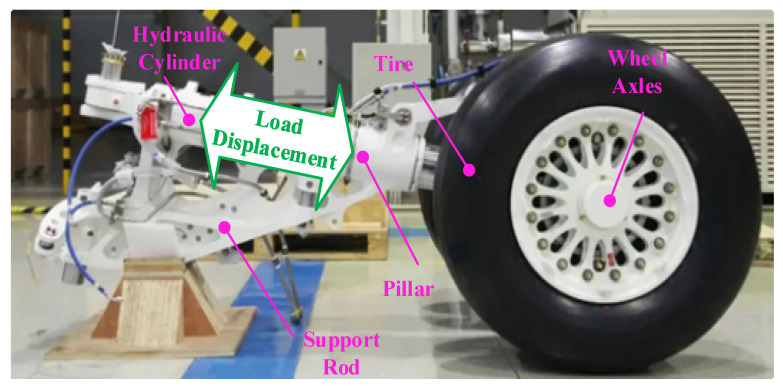
Landing gear structure composition.

Landing gear is the main support component for aircraft takeoff and landing, and its health status is closely related to aircraft flight safety [1,2,3]. As shown in Figure 1, the landing gear is mainly composed of a hydraulic cylinder, tire, wheel axles, pillar, support rod, and other components. In the aircraft takeoff and landing task, the landing gear is often subjected to large impacts due to its complex structural composition, resulting in the mechanical failure of landing gear components. In addition, the external load is prone to extreme peaks due to the changeable working conditions, resulting in performance degradation of the landing gear. Therefore, it is necessary to evaluate the takeoff and landing performance of the landing gear by combining working condition parameters and structural parameters to ensure the safety of aircraft operation. Establishing an accurate landing gear performance prediction model can not only provide a basis for structure setting and optimization of safety margin setting by fully considering the external load variation in the design phase, but also provide technical support for safety maintenance in the operation and maintenance phase by being able to fully consider the specific situation. Nevertheless, there is a complex nonlinear relationship between structure parameters, working condition parameters, and landing gear performance [4,5,6], which makes it very difficult to establish an accurate performance prediction model.

CAE (computer-aided engineering) is one of the commonly used methods for landing gear performance analysis. By steps such as meshing, boundary constraints, material definition, and iterative calculation, CAE can achieve precise static and dynamic analysis [7,8,9]. However, this method has certain limitations. On the one hand, CAE requires extensive iterative calculations, resulting in slow solving speed and long response time. On the other hand, CAE is a deterministic modeling method, which means that both structural parameters and working conditions are treated as fixed parameters during the calculation process. However, during long-term operations, working conditions are random, and structural parameters may slightly change (such as wear and tear).

To address the shortcomings of real-time and poor adaptability of CAE, some researchers have employed machine learning as the agent model for CAE. This is training machine learning models by CAE data or sensor data, and deploying machine learning models in real maintenance to achieve fast and accurate predictions. Learning-based methods have been developed for the performance prediction of complex industrial equipment [10,11,12]. Zhang et al. [13] proposed a vibration signal fault diagnosis method based on MLPC-CNN (convolution neural network based on multilayer pooling classifier), which uses an improved convolutional neural network for feature extraction, and a classifier for fault identification. Yan et al. [14] proposed a gear RUL prediction model based on LSTM (long short-term memory networks), which improved prediction accuracy and robustness by combining the tree structure with LSTM. Lan et al. [15] proposed a cavitation detection model based on MLP-Mixer (multilayer perceptron), which is used to recognize the cavitation intensity of the axial piston pump with given working conditions. Zhou et al. [16] proposed an improved SVM (support vector machine), which was optimized by the BAS (beetle antennae search) algorithm and PSO (particle swarm optimization) algorithm to achieve high-precision classification of ultrasonic signals. Dhiman H S et al. [17] proposed an anomaly detection method of wind turbine gearboxes using TWSVM and adaptive threshold to achieve accurate performance. Qiang S et al. [18] proposed an online fault diagnosis method of wind turbine blades based on SVM, which can achieve the expected effect.

Although the machine learning model has made significant breakthroughs in predicting the performance of complex equipment in the industry, and has improved the prediction accuracy [19,20,21], its prediction performance heavily depends on the training data. Thus, when using the machine learning model for landing gear performance prediction, some problems still need to be solved.

First of all, the actual collected samples of landing gear are high-dimensional, which would limit the computational efficiency and prediction accuracy of the machine learning model. After analysis with experts, the number of the monitoring parameters of the landing gear related to takeoff and landing performance is 15, including 11 working condition parameters (X1~X11), 4 structural parameters (X12~X15), and 2 performance parameters (Y1~Y2), as shown in Table 1. High-dimensional parameters would bring many difficulties to performance prediction based on machine learning. On the one hand, high-dimensional parameters contain some redundant information, i.e., some input parameters are highly correlated with each other. On the other hand, high-dimensional parameters contain invalid information, i.e., some input parameters have less influence on output results. Redundant information and invalid information will limit the calculation efficiency and prediction accuracy of the machine learning model. Thus, key features should be selected from high-dimensional parameters to reduce data dimensions and improve the calculation efficiency. However, in the case of incomplete knowledge of the mechanism, how to select a suitable dimension reduction method and ensure that the key features can effectively support performance prediction is a difficult problem faced by this paper.

Second, the structural parameters are time-varying and the working condition parameters of the landing gear are nonstationary, which leads to the distribution of collected monitoring data changing with the service time, making the deterministic modeling method not applicable. On the one hand, with the accumulation of service time and load impact, the structural parameters of the landing gear gradually change, so that the dynamic characteristics of the landing gear have also changed. The finite element model established previously has difficulty reflecting the actual state. On the other hand, due to the difference between the flight mission and the pilot’s operation behavior and different loads during flight, the working condition parameters of the landing gear are also changeable. The above reasons can lead to time-series changes in the distribution of landing gear state data, i.e., the distribution of data for the training model and the data used for prediction in the future may not be consistent. To improve the generalization of prediction models, some scholars fuse multiple single prediction models by integrated learning [22,23,24], which can improve the overall prediction accuracy under the complex data distribution. As a branch of machine learning, integrated learning can make full use of different base learners’ advantages. However, there are still some shortcomings in the existing integrated learning methods. For example, the dynamic weight assignment ability in the above work is weak, which is difficult to achieve adaptive adjustment according to changes in the data distribution. Therefore, to achieve high prediction accuracy under different structural parameters and working parameters, how to automatically assign each model’s weight according to the change of data distribution state is another difficult problem faced by this paper.

From the above analysis, to improve the prediction accuracy and generalization of the landing gear performance prediction model, two difficult problems should be focused on: (1) eliminate the redundant information and invalid information from the original landing gear performance parameters to realize the selection of key parameters, that is, feature selection; (2) adaptive sensing of the landing gear performance data distribution state caused by the gradually changeable structure and random loads, and achieve adaptive adjustment of the weights of each base model with the data distribution state by the weight adaptive learning mechanism, to reduce the prediction error caused by changes in distribution uncertainty.

To solve the above two difficult problems, a novel landing gear performance prediction method, the so-called MCA-MLPSA (multiple correlation analysis and MLP with self-attention) is proposed to improve the accuracy and generalization of landing gear performance prediction. First, to address the problem of feature selection, an MCA (multiple correlation analysis) method is proposed, which removes the invalid features that have little influence on the takeoff and landing performance by sensitivity analysis, and removes the redundant features in the original multiple parameters by redundancy analysis, so that the key features can be selected. Second, to adapt to the complex data distribution state, an integrated learning framework based on heterogeneous multiple learners is proposed, which integrates the selected multiple base model (i.e., the single machine learning models with high accuracy and large differences) into a framework to strengthen the generalization ability by making full use of each base model’s advantage. Third, to achieve the weight adaptive learning of the integrated learning framework, an MLPSA (MLP with self-attention) is proposed, which learns the complex data distribution by self-attention and adaptively assigns the weights of each base model by MLP (multilayer perceptron), so that the weight of integrated learning can be dynamically adjusted with the data distribution. Finally, the excellent prediction performance of the developed MCA-MLPSA is validated by a series of experiments on the landing gear takeoff and landing performance dataset. The key contributions of this paper are summarized as follows:

1. Aiming at the problems of high complexity, low calculation efficiency, and limited accuracy of the prediction model caused by the high-dimension parameters of landing gear, a multiple correlation analysis method is proposed for key features selection, which can effectively remove the invalid features and redundant features from the original high-dimension parameters by sensitivity analysis and redundancy analysis.
2. An integrated learning framework composed of heterogeneous base learners is proposed to solve the problem that the single model has a limited learning ability and difficulty applying to the complex data distribution.
3. Aiming at the problem of weight adaptively learning of integrated learning, a novel MLPSA (self-attention and MLP) is proposed, which introduces the self-attention mechanism into the MLP neural network to adaptively learn the correlation of multiple base learners’ predicted values, to strengthen the sensing ability of integrated learning for the data distribution state and realize adaptive weight adjustment.
4. Experiments show that the developed MCA-MLPSA can accurately predict landing gear performance in the case of different data distributions, and is superior to other integrated learning methods.

The rest of this paper is organized as follows: Section 2 introduces the motivation of this paper, and describes the principle, structure, and implementation of the proposed MCA-MLPSA in detail, and carefully explains how to achieve the weight adaptive learning of the integrated learning framework by self-attention. Section 3 presents the application and results of the proposed MCA-MLPSA on the landing gear takeoff and landing performance dataset, and provides a detailed analysis of the experimental results. The conclusions are given in Section 4.

## 2. Methodology

**Figure 2:**
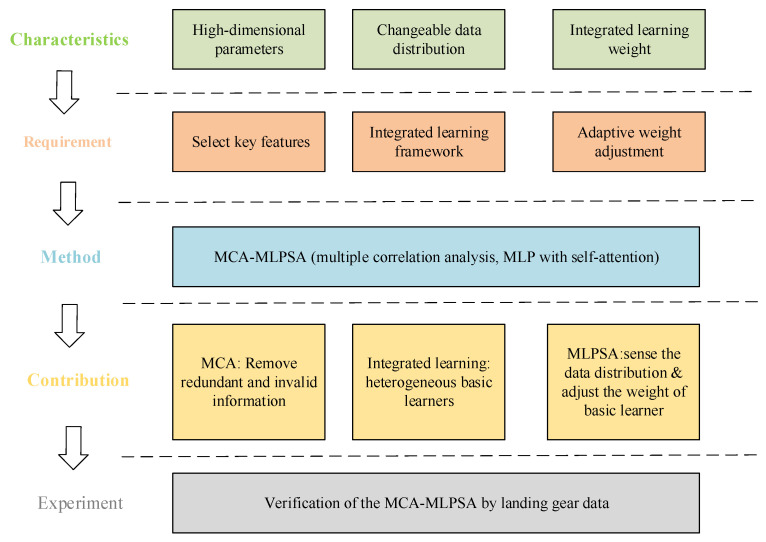
Research outline.

When predicting the performance of the landing gear, two inherent problems require solving: (1) The original parameters of the landing gear contain some redundant information and invalid information, which would seriously reduce the prediction efficiency and accuracy. (2) On the one hand, the structural parameters of the landing gear would change with the increase of service time (i.e., time gradient) due to factors such as wear of the components. On the other hand, the working conditions of the landing gear are complex and changeable, which would lead to a difference between the currently collected working condition parameters and the future working condition parameters (i.e., the uncertainty of the working condition). These two reasons would make the distribution of the collected landing gear monitoring data complex (i.e., the distribution of data used for training the model is different from that of the data used for prediction in the future), which severely limits the prediction effect of the established prediction model. To address these two difficult problems, this paper initially carefully analyzes the main characteristics of landing gear parameters, and then analyzes the actual requirements for performance prediction of the landing gear in detail, and finally designs an MCA-MLPSA for landing gear performance prediction. Figure 2 shows the research outline of this paper.

**Figure 3:**
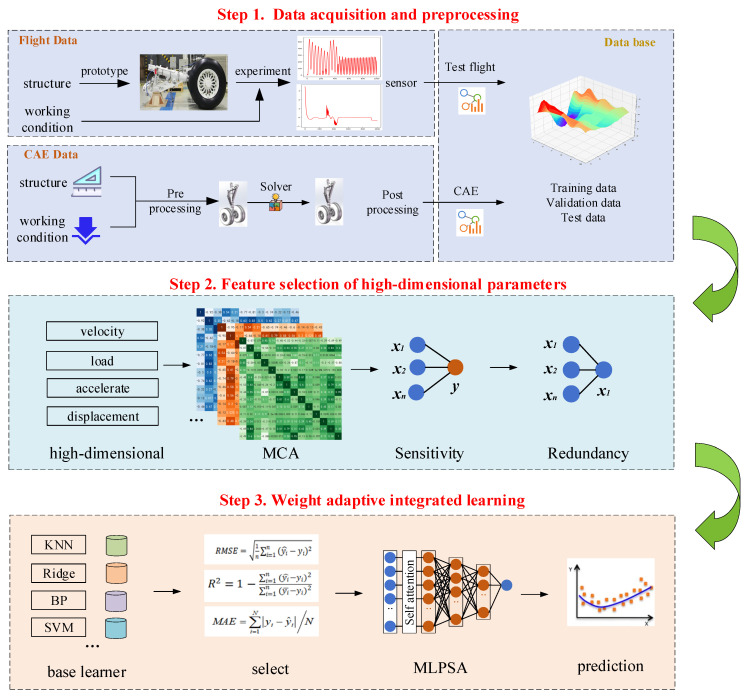
MCA-MLPSA model.

Figure 3 describes the schematic diagram of the proposed MCA-MLPSA, which mainly consists of three parts (i.e., step 1, step 2, step 3). Step1 is for data acquisition and preprocessing, step 2 mainly includes feature selection of high-dimensional parameters, and step 3 mainly includes weight adaptive integrated learning. In step 1, the input data are obtained from the performance monitoring sensor of the landing gear or CAE analysis. Before inputting them into MCA-MLPSA, these data will be normalized. The relevant contents will be carefully described in Section 4. In step 2, the MCA model is built for key feature selection. Through sensitivity analysis and redundancy analysis, key features that have a large impact on takeoff and landing performance and are mutually independent are selected. The relevant contents will be described in detail in Section 2.1. In step 3, the MLPSA model is proposed for weight adaptive learning. The weight of each base learner is adjusted through the self-attention mechanism, so that the weight assigned by integrated learning can dynamically adapt to the data distribution. The relevant contents will be described in detail in Section 2.2.

To remove the redundant information and invalid information from the original parameters of the landing gear as much as possible, a novel MCA is proposed to select the key features by making full use of sensitivity analysis and redundancy analysis.

**Figure 4:**
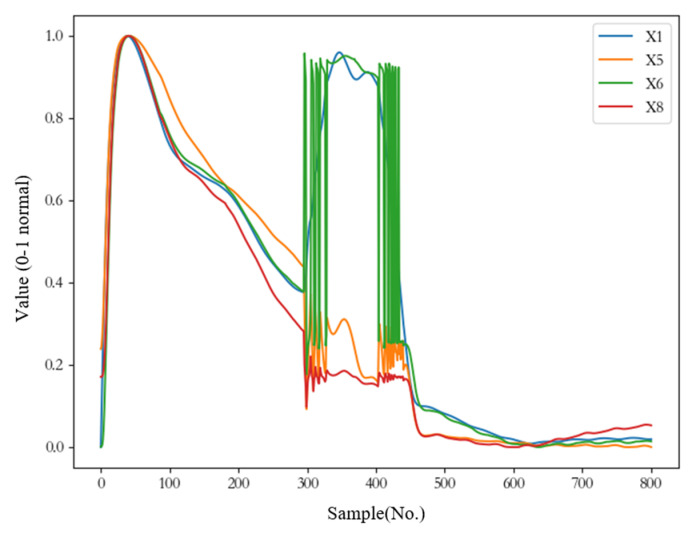
Changes of landing gear parameters.

As shown in Table 1, there are 15 landing gear parameters, but not all monitoring parameters provide useful information for performance prediction. Figure 4 visualizes some monitoring parameters. The horizontal coordinate is the sample size, that is, 800 samples collected during a landing process. The vertical coordinate is the changing trend of monitoring parameters (X1, X5, X6, X8), which has been normalized into 0–1. As shown in Figure 4, the changing trend of some parameters (such as X1, X5, X6, X8) is highly correlated, which indicates that some redundant information may exist, i.e., a parameter can be replaced by other parameters, such as X1 = f (X5, X6, X8). There is some linear or nonlinear relationship between X1 and X5, X6, and X8, which means X1 can be derived from X5, X6, and X8. In addition, the degree of influence of the above 15 parameters on the takeoff and landing performance is also different. Some parameters with little influence on takeoff and landing performance exist, which are regarded as invalid features in this paper. The existence of redundant information and invalid information would seriously limit the convergence speed and prediction accuracy of machine learning models. Thus, it is necessary to select key features to reduce the influence of redundant information and invalid information on the performance prediction of landing gear.

### 2.1. Feature Selection for Landing Gear Parameters via MCA

**Figure 5:**
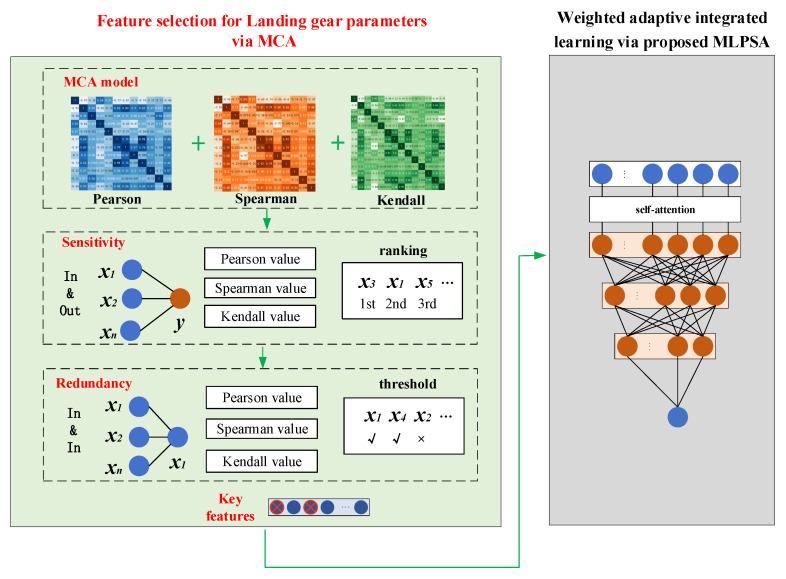
Structure of MCA model.

This paper proposes a novel landing gear parameter feature selection method, the so-called MCA, and the specific process is shown in Figure 5. First, the sensitivity of input parameters and takeoff and landing performance is analyzed, and the correlation coefficient between them is used to represent the impact of input parameters on takeoff and landing performance. The lower the correlation coefficient is, the weaker the influence of this input parameter on takeoff and landing performance is, which is then regarded as a candidate invalid feature. Second, the internal redundancy of the input parameters is analyzed, and the correlation coefficient between the two input parameters is also used to represent the correlation between them. A high correlation coefficient between the two input parameters indicates a strong correlation between them. The input parameters whose correlation coefficient is higher than the threshold value are regarded as candidate redundant features. After sensitivity analysis and redundancy analysis, the key features are selected.

The correlation coefficient methods used in sensitivity analysis and redundancy analysis mines the correlation between landing gear parameters based on statistics. Since the landing gear has complex structural components and working conditions, it is difficult to conduct a comprehensive analysis with a single correlation coefficient. 

To fully explore the linear, nonlinear, and rank correlation between high-dimension parameters, this paper proposes an MCA analysis method i.e., Pearson coefficient, Spearman coefficient, and Kendall coefficient are integrated to establish PSK indicators for redundancy analysis and sensitivity analysis. The calculation of PSK is shown in Equations (1) and (2):

(1) PSK(red)=Xrp(xi,xj)∪Xrs(xi,xj)∪Xrk(xi,xj)

(2) PSK(sen)=Xsp(x,y)∩Xss(x,y)∩Xsk(x,y)

PSK(red) represents the result of redundancy analysis, while Xrp, Xrs, and Xrk represent the correlation between input parameter xi and xj using the Pearson coefficient, Spearman coefficient, and Kendall coefficient, respectively. When any correlation (i.e., Xrp or Xrs or Xrk) between input parameter xi and input parameter xj is above the threshold, the input parameter xi and xj are regarded as redundant features.

PSK(sen) represents the result of sensitivity analysis, while Xsp, Xss, and Xsk represent the correlation between input parameter x and output parameter y using the Pearson coefficient, Spearman coefficient, and Kendall coefficient, respectively. When all correlations (i.e., Xsp and Xss and Xsk) between input parameter x and output parameter y are below the TOP-K ranking, the input parameter x is regarded as an invalid feature.

Through PSK indicators, i.e., PSK(red) and PSK(sen), three correlation coefficients (Pearson, Spearman, Kendall) can be effectively unified. By complementing the advantages of different correlation coefficients, the MCA model is suitable for complex and variable data distribution, providing strong support for feature extraction of landing gear under different operating conditions.

The correlation coefficients (Pearson, Spearman, Kendall) used for Xrp, Xrs, Xrk, Xsp, Xss, Xsk will be detailed in Equations (3)–(6).

The Pearson coefficient is suitable for continuous data with normal distribution, which is used to calculate the linear correlation between landing gear parameters. The calculation formula of the Pearson coefficient is given in Equation (3)

(3) $$r_{p}\left(x,y\right)=\frac{cov\left(x,y\right)}{\sigma_{x}\sigma_{y}}$$

where $r_{p}\left(x,y\right)$ is the Pearson correlation of parameter x and parameter y. $\sigma_{x}$ is the standard deviation of the parameter x;$\;\sigma_{y}$ is the standard deviation of the parameter y; and cov is the covariance.

The Spearman coefficient is applicable to continuous data with non-normal distribution, and can be used to calculate the nonlinear correlation between landing gear parameters. The calculation formula of the Pearson coefficient is given in Equations (4) and (5)

(4) $$r_{s}\left(x,y\right)=1-\frac{6\sum d_{i}^{2}}{n\left(n^{2}-1\right)}$$

(5) $$d_{i}=r_{g}\left(x_{i}\right)-r_{g}\left(y_{i}\right)$$

where $r_{s}\left(x,y\right)$ is the correlation between parameter x and parameter y, n is the amount of data, $d_{i}$ is the difference between the two data orders, and $r_{g}$ is the data order.

The Kendall coefficient is applicable to orderly data and can be used to calculate the level correlation between landing gear parameters. The calculation formula of the Pearson coefficient is given in Equation (6)

(6) $$r_{k}=\frac{\left(N_{1}-N_{2}\right)}{\sqrt{\left(N_{0}-N_{3}\right)\times \left(N_{0}-N_{4}\right)}}$$

where $N_{0}$ is the total number of parameter pairs, $N_{1}$ is the number of positively correlated parameter pairs, $N_{2}$ is the number of negatively correlated parameter pairs, $N_{3}$ is the number of parameters with equal values in *x*, and $N_{4}$ is the number of parameters with equal values in *y*. 

For sensitivity analysis, PSK(sen) is used to filter invalid features. By using PSK(sen) indicator, the influence of every input parameter is calculated and ranked. Then, using TOP-K strategy for truncation, the input parameters in the TOP-K ranking should be retained, which are the key features and have a greater influence on the output. The input parameters after TOP-K ranking are regarded as invalid features, because they have little influence on the output. By the sensitivity analysis and TOP-K strategy, the invalid features can be removed.

For redundancy analysis, PSK(red) is used to filter redundant features. By using PSK(red) indicator, the redundancy between two input parameters is calculated. If the redundancy between two input parameters is above the threshold, one of them will be regarded as a redundant feature and will be removed. 

Through PSK indicator, i.e., PSK(sen) and PSK(red), sensitivity analysis and redundancy analysis are performed. The key features that have a greater impact on landing gear performance and are independent from each other are selected for integrated learning prediction.

### 2.2. Weighted Adaptive Integrated Learning via Proposed MLPSA

**Figure 6:**
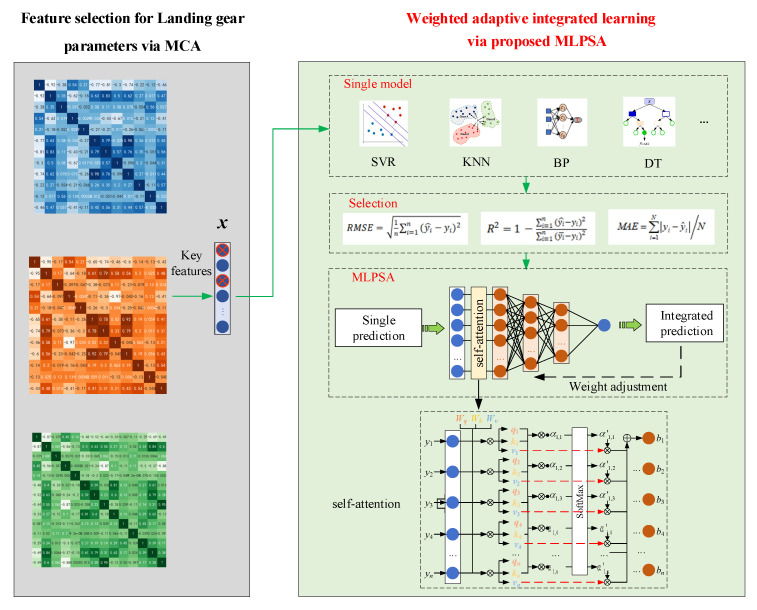
Structure of MLPSA model.

To solve the problem of adaptive weight assignment of integrated learning, a novel MLPSA is proposed to dynamically change the weights of base learners by the self-attention mechanism. Due to the time gradient of structural parameters and uncertain working condition parameters of the landing gear, the distribution of monitoring data changes during service time. However, a single model has limited ability for learning data in a complex distribution. Meanwhile, the conventional integrated learning uses fixed weight assignment, which is difficult to achieve accurate prediction. To this end, a novel integrated learning model, the so-called MLPSA, is proposed, which can dynamically adjust the weights of base learners with data distribution. As shown in Figure 6, the developed MLPSA consists of base learners and MLP with self-attention. The base learners are selected from single models and used for predicting landing gear performance. The self-attention is used for sensing data distribution, and MLP is used for adjusting the weight of each base learner. The specific process is described below. First, single models with high accuracy are selected as base learners, which use the key features selected by MCA as input. Then, the self-attention is used for sensing the value sequences consisting of the predicted values of all base learners. The MLP with self-attention is used for learning the data distribution of value sequences, which can pay more attention to the prediction value with high correlation, so the final result can shift toward the core of the value sequences.

The base learners are selected based on the principle of “good but different”, which are selected from 7 different common prediction models, including: Ridge, ElasticNet, KNN, SVM, DT, BP, and RBF. Through the differences in data observation and training modes of different base learners, integrated learning composed of heterogeneous base learners can break the limitation of single models, which is beneficial to sense complex data distribution and learn the implicit mapping relationship. Ridge is a regression model, which solves the overfitting in the training process. ElasticNet is an elastic network model, which strengthens the sparsity and generalization. KNN is a K-nearest neighbor model, which measures the samples by distance to judge the current sample. SVM divides the samples in the feature space by maximizing the margin, and usually has strong robustness. DT is a decision tree model, which summarizes the decision rules and constructs a tree structure for prediction, and has a strong mechanism explanation. BP is a fully connected neural network, which adjusts the weights of neurons through reverse gradient propagation, and has strong fitting ability for nonlinear systems. RBF is a three-layer feed-forward neural network, which has a simple structure and fast learning speed. It can be seen that the conventional machine learning models, such as Ridge, ElasticNet, KNN, SVM, DT, BP, and RBF, are different in composition structure, prediction mechanism, and learning method.

Nevertheless, the applicability and accuracy of the above single models still need to be evaluated on the data collected from landing gear. Thus, this paper uses three metrics (i.e., MAE, MAPE, and HAPE) to evaluate these single models, and those single models with high accuracy are selected as base learners. The calculation of MAE, MAPE, and HAPE is given in Equations (7)–(9).

(7) $$\mathrm{MAE}=\sum_{i=1}^{M}\left|y_{i}-\hat{y_{i}}\right|/M$$

(8) $$\mathrm{MAPE}=\sum_{i=1}^{M}\frac{\left|y_{i}-\hat{y_{i}}\right|}{y_{i}}/M\times 100\%$$

(9) $$\mathrm{HAPE}=\left[\min\left(\frac{\left|y_{i}-\hat{y_{i}}\right|}{y_{i}}\right),\max\left(\frac{\left|y_{i}-\hat{y_{i}}\right|}{y_{i}}\right)\right],i\in \left[1,M\right]$$

where *M* is the total number of samples, *i* is the current sample, $y_{i}$ is the real value, and $\hat{y_{i}}$ is the predicted value. MAE is mean absolute error, MAPE is mean absolute percentage error, and HAPE is the horizon of absolute percentage error.

The N base learners (N < 7) with high accuracy are selected by MAE, MAPE, and HAPE. Based on high-precision single models, the integrated learning idea is used to fuse the N prediction results to obtain the final output, which can improve the overall accuracy and generalization. In the process of integrated learning, due to the different structures and learning modes, each base learner’s initial prediction is also different. In theory, the initial predictions from multiple base learners can be regarded as sequence information, which will change dynamically with the distribution of landing gear parameters. Nevertheless, conventional integrated learning, whether using fixed weight assignment or using weight learning, ignores analyzing the sequence information. The sequence information obtained from base learners is strongly related to the distribution of the data collected from landing gear. The changes of the sequence information should be analyzed carefully, and the weight distribution should be adjusted adaptively, to make prediction more accurate.

**Figure 7:**
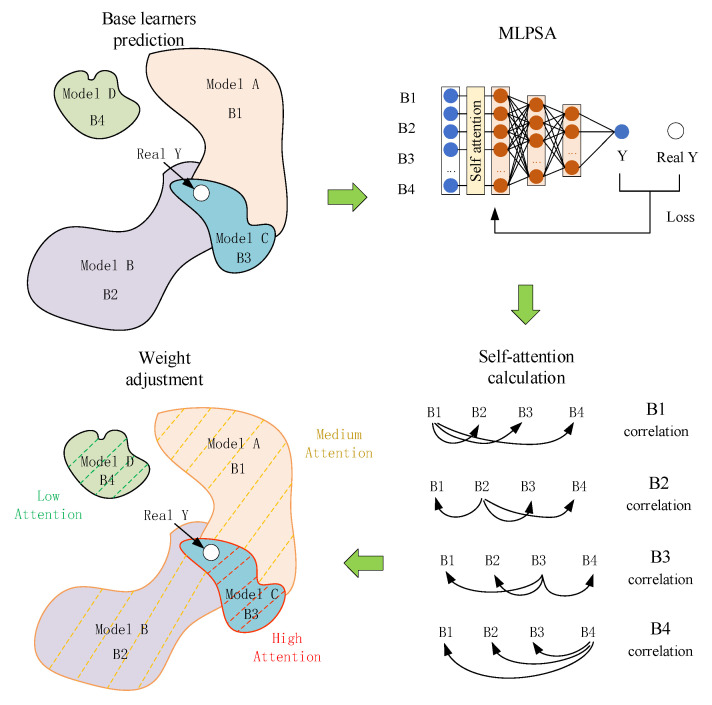
Principle of weight adaptive integration.

To solve this problem, a weight adaptive integration method, the so-called MLPSA, is proposed. MLPSA combines MLP with self-attention to sense the sequence information obtained from base learners, and adjust the weight of each base learner adaptively. Specifically, the prediction with the highest correlation with others is discovered, and assigned more attention. Then, the weights of all base learners are adaptively changed according to the correlation, so that the final result of integrated learning is close to the real value, as shown in Figure 7.

MLPSA takes the selected key features as input, and obtains the prediction value sequence {B1, B2, B3 … Bn} by using multiple single machine learning models. When the data distribution of the landing gear parameter changes, the sequence information also changes. To effectively sense this change, MLPSA extracts the internal correlation in the sequence information through self-attention. This takes the prediction value with the highest correlation with others as the dominant value and adaptively adjusts the weight of each base learner according to the correlation. In the integrated learning process, the base learners produce a variety of predictions for landing gear performance. The correlation between different predictions is calculated through the matrix called q, k, v, so that the final prediction value is fused based on correlation. Specifically, the q and k matrices are multiplication for obtaining the internal correlation in sequence information. The results are scaled based on dimension d for reducing the computation. The softmax layer is used for weight conversion, and multiplied with the v matrix to output the correlation, which is calculated in Equation (10).

(10) $$\mathrm{Attention}=\mathrm{softmax}\left(\frac{{\mathrm{qk}}^{T}}{\sqrt{d_{k}}}\right)v$$

### 2.3. Flowchart of MCA-MLPSA Landing Gear Performance Prediction 

**Figure 8:**
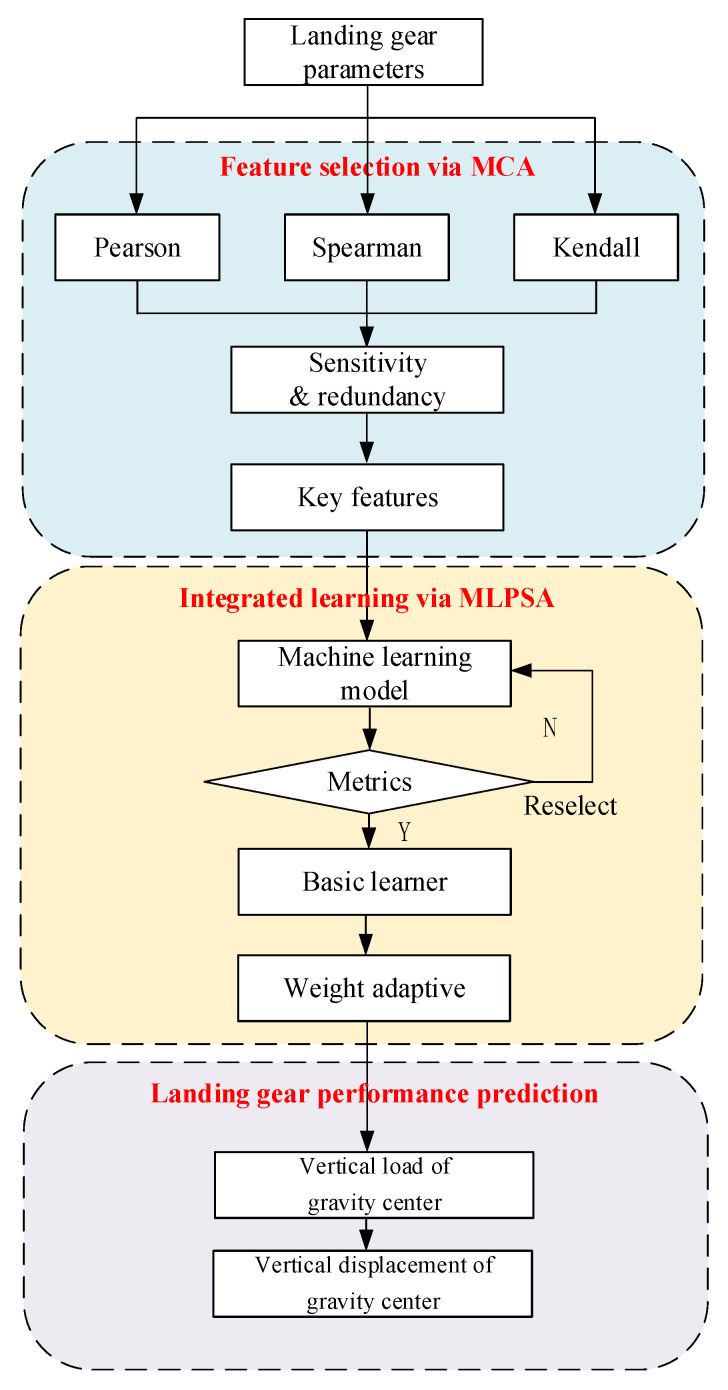
Landing gear performance prediction process.

Figure 8 describes the flowchart of using the proposed MCA-MLPSA to predict the landing gear performance. First, the landing gear parameters are collected by the sensors and normalized, and the proposed MCA is used for sensitivity analysis and redundancy analysis. Second, take the selected key features as input, and 7 machine learning models (i.e., Ridge, Elastic, KNN, SVM, DT, BP, RBF) are used to predict the landing gear performance; those machine learning models with higher accuracy are selected as base learners, which are used to form the integrated learning framework for final prediction. Third, the weights of base learners are adaptively adjusted by the proposed MLPSA. Finally, the trained MCA-MLPSA is used to predict the landing gear performance (i.e., the vertical load of gravity center, the vertical displacement of gravity center).

## 3. Experiment

### 3.1. Description of Experiment Data

The proposed MCA-MLPSA is applied to a landing gear performance dataset to prove its effectiveness. The dataset includes 17018 samples. Each sample contains 15 input parameters (11 working condition parameters (X1~X11) and 4 structural parameters (X12~X15)) and 2 output parameters (Y1~Y2); the physical meanings of all parameters are described in Table 1. In addition, all parameters are discrete variables, including air pressure, mass, displacement, velocity, load, force, etc.

Before inputting these samples into the MCA-MLPSA, they will be normalized to eliminate the magnitude differences. The normalized samples are shown in Table 2. In the prediction process, X1–X15 are taken as input parameters, and Y1 (vertical load of gravity center) and Y2 (vertical displacement of gravity center) are treated as output parameters, respectively.

### 3.2. Analysis of Feature Selection

The dimension of landing gear parameters is high, and there are 15 parameters in total, including structural parameters and working condition parameters. The dimension of working condition parameters is 11, which is much more than structural parameters. Meanwhile, the operation mechanism of landing gear is complex, which leads to redundant information and invalid information among the working condition parameters. In contrast, there are only four structural parameters, which are few and designed by experts, and the physical correlation of the four structural parameters is weak. Therefore, this paper mainly focuses on the working condition parameters for feature selection.

#### 3.2.1. Sensitivity Analysis

First, sensitivity analysis is performed between the working condition parameters (X1–X11) and the output (Y1, Y2), as shown in Table 3 and Table 4. The ranking represents the influence degree of the input features on the output. The larger the value is, the higher the ranking is, and the greater influence is. The features with lower ranking are regarded as invalid information.

As shown in Table 3, for Y1 prediction (vertical load of gravity center), feature Pearson value, Spearman value, and Kendall values of X2, X4, X9, and X10 are very small and the corresponding rankings are low, which indicates that these input features have a weak influence on Y1. Therefore, X2, X4, X9, and X10 are regarded as the result of PSK(sen), i.e., invalid features. As shown in Table 4, for Y2 prediction (vertical displacement of gravity center), the Pearson value, Spearman value, and Kendall values of the input features X4, X7, X8, and X10 are very small and the corresponding rankings are low, which indicates that they have a weak influence on Y2. So, features X4, X7, X8, and X10 are regarded as invalid information in temporary. By sensitivity analysis, some invalid features corresponding to different outputs would be discriminated, and they will be treated as the candidates which will be removed.

#### 3.2.2. Redundancy Analysis

**Figure 9:**
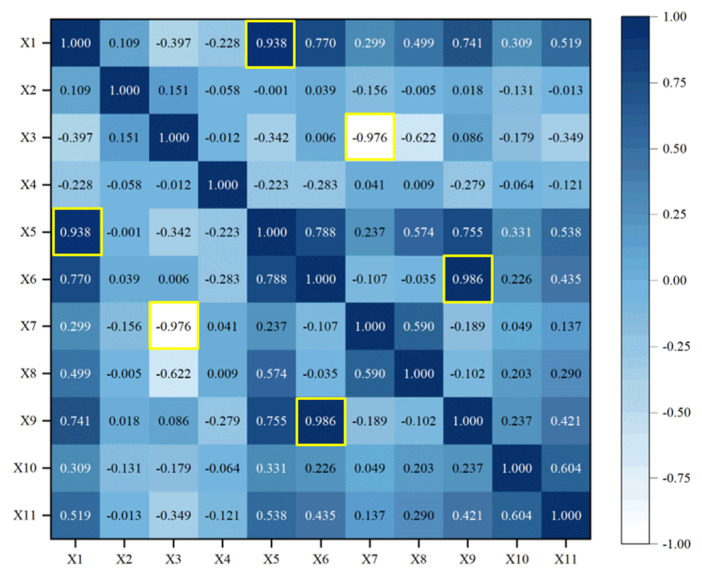
Pearson redundancy analysis.

**Figure 10:**
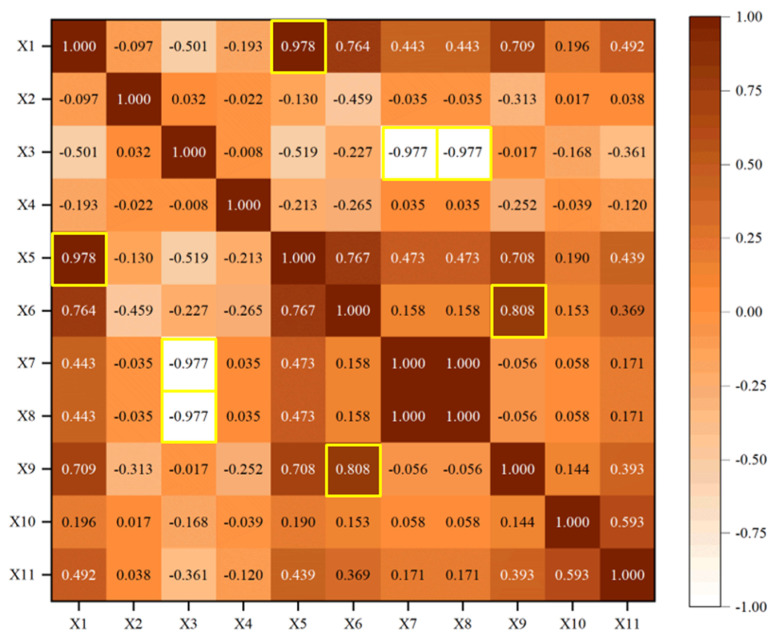
Spearman redundancy analysis.

**Figure 11:**
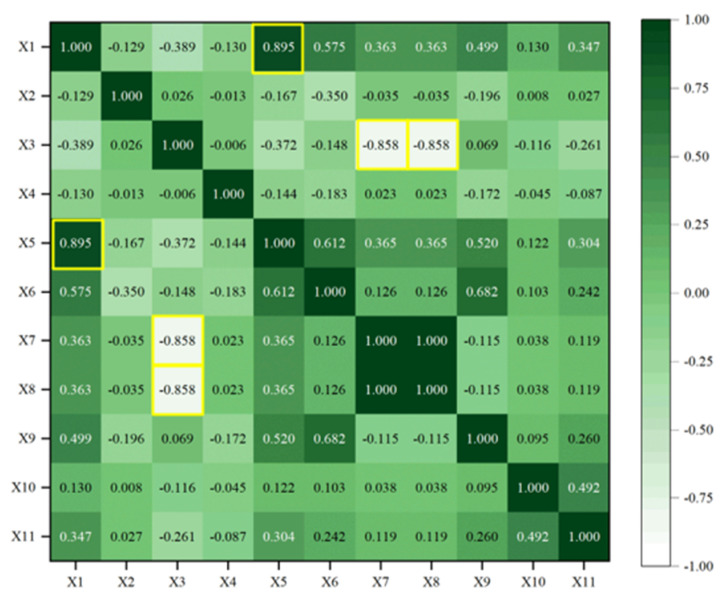
Kendall redundancy analysis.

After the sensitivity analysis, invalid information in the input parameters can be effectively extracted. Nevertheless, there is still some redundant information between input parameters. To further compress data dimensions, redundancy analysis needs to be carried out, i.e., Pearson, Spearman, and Kendall correlation coefficients are used to calculate the relationship in different input parameters. The larger the value is, the stronger the correlation is, that is, there are highly coupled redundant. The results of redundancy analysis in 11 working condition parameters are shown in Figure 9, Figure 10 and Figure 11.

As shown in Figure 9, Figure 10 and Figure 11, the values of the diagonal region are 1 (represented by dark), which indicates that the feature is highly correlated with itself. In addition, there are some dark regions (values close to 1) or white regions (values close to −1), which indicate that they are redundant features, i.e., they have strong positive/negative correlation with others. The redundant features (marked in yellow) in Figure 9, Figure 10 and Figure 11 are counted, as shown in Table 5.

It can be seen that there is strong linear, nonlinear, and rank redundancy between X1 and X5, X3, and X7. There is strong nonlinear and rank redundancy between X3 and X8. There is strong linear and nonlinear redundancy between X6 and X9. Therefore, (X1–X5), (X3–X7), (X3–X8), and (X6–X9) are regarded as the result of PSK(red), i.e., redundant feature groups.

From the analysis of Table 3 and Table 5, it can be seen that the invalid features are X2, X4, X9, and X10, and the redundant feature groups are (X1–X5), (X3–X7), (X3–X8), and (X6–X9). By comparing every two parameters in the redundant feature groups, X3 and X5 have less influence on Y1. Therefore, for Y1 prediction, invalid features X2, X4, X9, and X10, and redundant features X3 and X5 should be removed. The remaining features are the key features, which are independent from each other and have a great influence on Y1.

From Table 4 and Table 5, it can be seen that the invalid features are X4, X7, X8, and X10, and the redundant feature groups are (X1–X5), (X3–X7), (X3–X8), and (X6–X9). By comparing every two parameters in redundant feature groups, it is found that X5 and X9 have less impact on Y2. Therefore, for Y2 prediction, invalid features X4, X7, X8, and X10, and redundant features X5 and X9 should be removed. The remaining features are the key features, which are independent from each other and have a great influence on Y2. 

### 3.3. Analysis of Landing Gear Performance Prediction 

In this section, the corresponding selected key features are used as input for MLPSA to predict Y1 (vertical load of center of gravity) and Y2 (vertical displacement of center of gravity). To ensure the accuracy and flexibility of the predictions, this study constructed MLPSA for Y1 and Y2 separately. On the one hand, by keeping feature selection and base learner selection separate for Y1 and Y2, it is advantageous to select the most effective feature information and learning method for each. On the other hand, training Y1 and Y2 separately allows for adjustments and updates to be made to a single model (either Y1 or Y2) based on engineering requirements.

During the experiment, the landing gear data are randomly split with the proportion of 6:2:2, that is, 60% for the training set (containing 10210 samples), 20% for the validation set (containing 3404 samples), and 20% for the test set (containing 3404 samples). The training set is used to train the internal parameters of the model, the validation set is used to adjust hyperparameters, and the test set is used to evaluate the final prediction effect of the model. The prediction result is evaluated by three common metrics, i.e., MAE, MAPE, and HAPE.

#### 3.3.1. Base Learner Selection

The proposed integrated learning framework in this paper is composed of multiple heterogeneous base learners, so that the prediction accuracy of each base learner has a great influence on the final prediction. To this end, single models with high accuracy are selected as the base learners. In this paper, Ridge, Elastic, KNN, SVM, DT, BP, and RBF are constructed to predict Y1 (vertical load of center of gravity) and Y2 (vertical displacement of center of gravity). Then, according to the accuracy of each single model, the base learners are selected to realize the integrated learning.

Since hyperparameters will affect the feature learning and performance prediction, to compare different models more objectively, the grid search method is used to adjust the key hyperparameters of each model (Ridge, Elastic, KNN, SVM, DT, BP, RBF). The hyperparameter settings are shown in Table 6 and Table 7. The alpha of Ridge represents the L1 regularization coefficient. The l1_Ratio of Elastic represents the combined coefficient of L1 regularization and L2 regularization. The n_neighbors of KNN represents the number of samples nearby used to the judge current sample. The kernel and C of SVM represent the kernel function type and penalty factor, respectively. The criterion and max_depth of DT represent the basis for node splitting and the depth of the decision tree, respectively. The hidden_size and learning_rate of BP represent the hidden neurons and learning rate, respectively. The hidden_size and learning_rate of RBF represent the hidden neurons and learning rate, respectively.

The single model after hyperparameter optimization is applied to prediction of landing gear performance, and three metrics (MAE, MAPE, HAPE) are used to evaluated the prediction results. To avoid randomness, each experiment is repeated 10 times, and the averaged values are taken as the final results, as shown in Table 8 and Table 9.

For the prediction of Y1 (vertical load of gravity center), Elastic, KNN, SVM, and DT have lower values of MAE, MAPE, and HAPE, indicating better prediction results. For the prediction of Y2 (vertical displacement of gravity center), Ridge, KNN, SVM, and DT have lower values of MAE, MAPE, and HAPE, indicating better prediction results. From the analysis of the prediction results, the above base learners maintain a high accuracy when the data distribution of the landing gear is complex. 

Therefore, for Y1 prediction (vertical load of gravity center), Elastic, KNN, SVM, and DT are selected as the base learners. For Y2 prediction (vertical displacement of gravity center), Ridge, KNN, SVM, and DT are selected as the base learners. The base learners with high accuracy and different structures are initially used for predicting landing gear performance, and later providing a good basis for the integrated learning.

#### 3.3.2. The Prognostic Results of Weighted Adaptive Integrated Learning

Through the above analysis, we have selected base learners for Y1 prediction (vertical load of center of gravity) and Y2 prediction (vertical displacement of center of gravity). To further improve the prediction accuracy of integrated learning, MLPSA is proposed to sense the data distribution and adaptively adjust the weight of each base learner. The hyperparameters of the MLPSA are confirmed by the grid search method, as shown in Table 10 and Table 11. Layer1–Layer5 is the network layer, which is composed of Self Attention, Dense, Leaky_Relu. Optimizer is Adam, Lr is the learning rate, and Batchsize is the sample size of each batch.

To verify the rationality and superiority of MLPSA, the MLPSA is applied to the Y1 prediction and Y2 prediction, and compared with three other mainstream integrated learning methods (i.e., bagging, linear, MLP). Bagging is the weighted average method, which sums the predicted values of base learners to obtain the mean value. Linear is a linear regression layer, which fuses the predicted values of base learners in a linear way. MLP is a multilayer perceptron, which fuses the predicted values of base learners in a nonlinear way. The hyperparameters of the above integrated learning methods are confirmed by the grid search method. In addition, each group of experiments is repeated 10 times and the averaged value is taken as the final result, as shown in Table 12 and Table 13.

As depicted in Table 12, compared with three other integrated learning methods (bagging, linear, MLP), the developed MLPSA decreases the MAE by 38.378%, 44.118%, and 25.490%, respectively, decreases the MAPE by 23.248%, 41.363%, and 28.487%, respectively, and has a better performance on the HAPE.

In addition, as can be seen from the prediction results in Table 7 and Table 12, compared with seven single models (Ridge, Elastic, KNN, SVM, DT, BP, RBF), the developed MLPSA decreases the MAE by 80.379%, 73.793%, 49.333%, 74.497%, 34.104%, 78.244%, and 82.569%, respectively, decreases the MAPE by 76.280%, 68.943%, 46.916%, 62.226%, 54.953%, 69.799%, and 80.230%, respectively, and has a better performance on the HAPE. 

**Figure 12:**
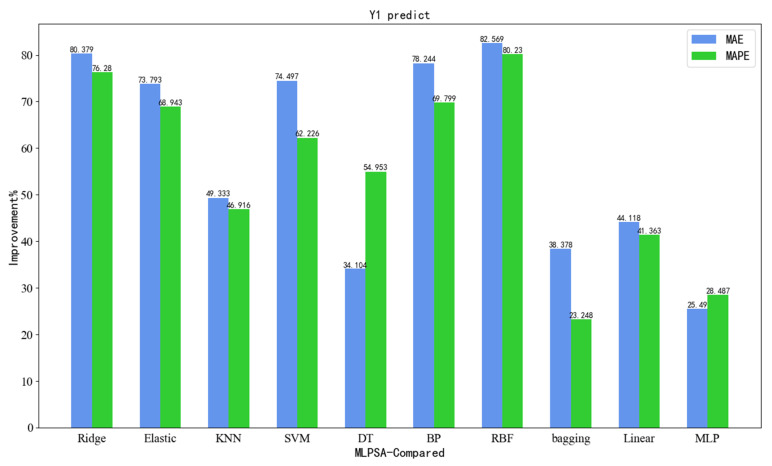
Regression accuracy improvement (Y1).

To visualize the superiority of the developed MLPSA, Figure 12 illustrates the accuracy comparison results presented in the form of bar graphs. It can be seen that the developed MLPSA has achieved the best prediction results in Y1 (vertical load of gravity center). Compared with single models, MLPSA has significant advantages in regression metrics such as MAE and MAPE. Meanwhile, the developed MLPSA has a better prediction performance than that of other integrated learning methods.

As shown in Table 13, compared with three integrated learning methods (bagging, linear, MLP), the MLPSA decreases the MAE by 44.828%, 49.474%, and 28.889%, respectively, decreases the MAPE by 32.374%, 58.862%, and 25.100%, respectively, and has a better performance on the HAPE.

In addition, as can be seen from the prediction results in Table 9 and Table 13, compared with seven single machine learning models (Ridge, Elastic, KNN, SVM, DT, BP, RBF), the MLPSA decreases the MAE by 78.523%, 82.796%, 51.020%, 74.869%, 60.976%, 83.533%, and 86.536%, respectively, and decreases the MAPE by 77.672%, 80.894%, 64.122%, 67.530%, 69.529%, 78.612%, and 81.870%, respectively. 

**Figure 13:**
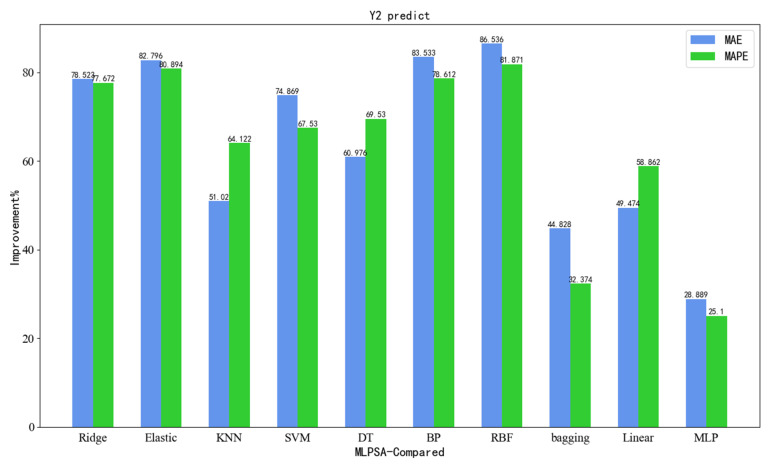
Regression accuracy improvement (Y2).

To visualize the superiority of MLPSA, Figure 13 also shows the above accuracy comparison results in the form of bar graphs. It can be seen that the developed MLPSA has achieved the best prediction effect in Y2 (vertical displacement of gravity center). Compared with single models and conventional integrated learning methods, the proposed MLPSA has significant advantages in three metrics (i.e., MAE, MAPE, and HAPE).

From the above analysis, compared with single models, integrated learning has achieved better prediction results. This is because integrated learning can complement itself through the differences in data observation and training modes of each base learner, making up for the limitations of a single model. It is worth noting that compared with other integrated learning methods, the developed MLPSA achieves the best prediction results. This is due to the self-attention mechanism, which makes the weight distribution of integrated learning more reasonable, i.e., dynamically adjusting the weight of each base learner with the distribution of data.

In the scenario of gradually changeable structure and random loads, the landing gear performance data have distribution differences, i.e., the distribution of training data and test data is different, which makes it difficult for the single model or traditional integrated learning to achieve an accurate prediction of landing gear performance. The developed MLPSA improves the accuracy of takeoff and landing performance prediction through adaptive weight adjustment, which is of great significance in engineering practice. On the one hand, it helps to optimize the parameters in the design phase, giving consideration to the safety threshold. On the other hand, it helps to intelligently monitor the operation and maintenance stages to ensure flight safety.

**Figure 14:**
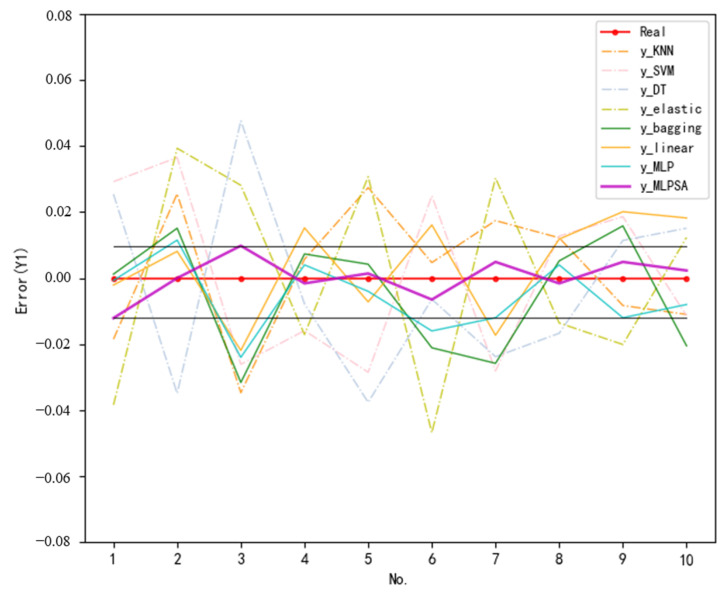
Prediction error (Y1).

**Figure 15:**
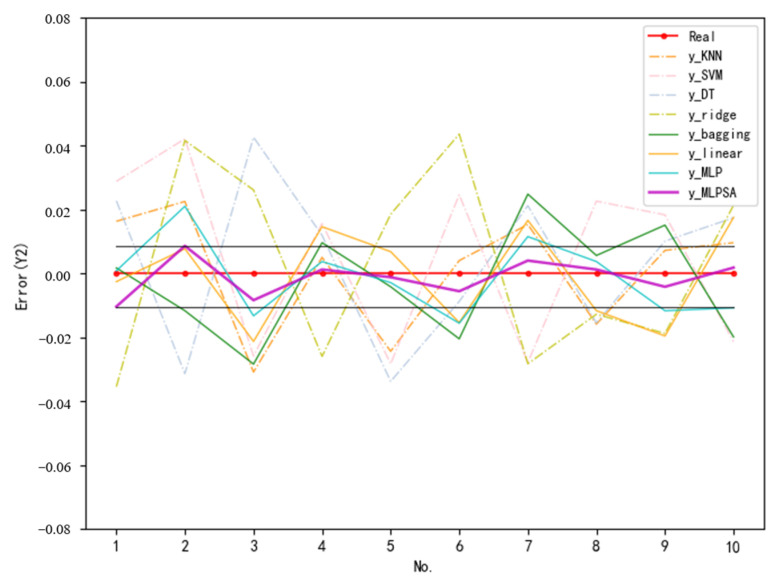
Prediction error (Y2).

To further demonstrate the excellent prediction ability of MLPSA, the prediction values and actual values of some samples randomly selected from the test set are visualized. Figure 14 illustrates the prediction errors of various methods for Y1 (vertical load of gravity center), while Figure 15 shows the prediction errors of various methods for Y2 (vertical displacement of gravity center). In Figure 14 and Figure 15, the horizontal coordinates are the no. of sample points and the vertical coordinates are the normalized error. The dashed lines are the prediction errors of single machine learning models (KNN, SVM, DT, Elastic, Ridge), while the solid lines are the prediction errors of the integrated learning models (bagging, Linear, MLP, MLPSA).

No matter the prediction for Y1 or Y2, the prediction error curves of the single models fluctuate greatly within the ±0.05 interval, while the prediction error curves of integrated learning (solid line legend) fluctuate within the ±0.03 interval. The experimental results indicate that integrated learning can complement the limitations of a single model by the advantages of multiple models, thus reducing the prediction error. More importantly, compared with three other integrated learning models, the MLPSA (purple solid line) has the smallest curve fluctuation within the ±0.012 interval (fluctuation range is marked with a straight line), which is closest to the 0 error line. Thus, the experimental results prove again that the developed MLPSA can substantially reduce the prediction error through weight adaptation, and achieve high-accuracy landing gear performance prediction.

## 4. Conclusions

Improving the performance prediction accuracy of aircraft landing gear has been a difficult issue in engineering practice. On the one hand, because the performance parameters of the landing gear include many structural parameters and working condition parameters, excessive redundant parameters and invalid parameters limit the prediction efficiency. On the other hand, the time-asymptotic nature of the structural parameters and the uncertainty of the working condition parameters severely limit the prediction accuracy of the prediction model. To solve this problem, a novel MCA-MLPSA model is proposed to accurately predict the performance of aircraft landing gear. Specifically, a novel MCA method is used to analyze the redundancy and sensitivity of high-dimensional parameters and select the key features. Then, the integrated learning framework, based on heterogeneous base learners, is adopted, which can realize the adaptive sense of landing gear performance data distribution states and adaptive assignment of weights of each base learner in the integrated learning framework by the introduction of a self-attention mechanism.

The effectiveness of the proposed MCA-MLPSA is verified by a series of experiments on the landing gear takeoff and landing performance dataset in comparison with three popular integrated learning models and seven single models. The experimental results show that the developed MCA-MLPSA can effectively reduce the data dimension by eliminating redundant features and invalid features through MCA (multiple correlation analysis), and substantially improve the prediction performance by weight adaptive learning. Compared with the seven single models (Ridge, Elastic, KNN, SVM, DT, BP, RBF) and three integrated learning models (bagging, Linear, MLP), the proposed MLPSA achieved the best prediction results in the MAE, MAPE, and HAPE. The above experimental results show that the proposed MCA-MLPSA in this paper can achieve accurate takeoff and landing performance prediction, which is of great significance for flight safety.

## Figures and Tables

**Table 1:** Input and output parameters.

X1	X2	X3	X4	X5	X6
Vertical displacement of tires	Vertical velocity of tires	Horizontal displacement of tires	Horizontal velocity of tires	Pillar velocity	Buffer load
X7	X8	X9	X10	X11	X12
Spring force	Damping force of fluid	Buffer Friction	Structure Force	Pillar force	Chamber I Pressure
X13	X14	X15	Y1	Y2	
Chamber II Pressure	Tire pressure	Weight	Vertical load of gravity center	Vertical displacement of gravity center	

**Table 2:** Normalized samples (partial).

No.	Input								Output	
	Working Condition Parameters					Structural Parameters			Performance	
	X1	X2	...	X10	X11	X12	...	X15	Y1	Y2
1	0.968	0.465	...	0.299	0.561	0.600	...	0.416	0.411	0.973
2	0.857	0.138	...	0.417	0.113	0.600	...	0.416	0.898	0.004
3	0.281	0.128	...	0.480	0.656	0.600	...	0.000	0.168	0.002
4	0.428	0.236	...	0.281	0.549	0.600	...	0.000	0.293	0.007
5	0.628	0.192	...	0.256	0.508	0.000	...	0.416	0.934	0.413
6	0.609	0.030	...	0.044	0.368	0.000	...	0.416	0.949	0.047
7	0.406	0.204	...	0.451	0.637	0.800	...	0.833	0.578	0.006
8	0.744	0.230	...	0.336	0.584	0.600	...	0.000	0.744	0.002
9	0.943	0.288	...	0.577	0.735	0.600	...	0.416	0.997	0.189
...	...	...	...	...	...	...	...	...	...	...
17018	0.750	0.179	...	0.551	0.703	0.000	...	0.416	0.784	0.538

**Table 3:** Y1 sensitivity analysis.

Feature	Pearson		Spearman		Kendall	
	Value	Ranking	Value	Ranking	Value	Ranking
X1	0.383079	4	0.494972	5	0.399855	4
**X2**	**0.141004**	**7**	**0.363665**	**9**	**0.293423**	**9**
X3	0.981946	2	0.980942	3	0.871405	3
**X4**	**0.018364**	**11**	**0.015115**	**10**	**0.010454**	**11**
X5	0.316543	5	0.522409	4	0.396595	5
X6	0.031670	10	0.215045	6	0.159058	6
X7	0.996032	1	0.997465	1	0.963418	1
X8	0.617989	3	0.997464	2	0.963391	2
**X9**	**0.113472**	**8**	**0.002074**	**11**	**0.081934**	**8**
**X10**	**0.076653**	**9**	**0.070595**	**8**	**0.046533**	**10**
X11	0.180979	6	0.200939	7	0.137894	7

Note: Bold underline means the result of PSK(sen).

**Table 4:** Y2 sensitivity analysis.

Feature	Pearson		Spearman		Kendall	
	Value	Ranking	Value	Ranking	Value	Ranking
X1	0.760885	3	0.863477	2	0.685512	2
X2	0.317166	6	0.200522	10	0.163976	10
X3	0.047498	10	0.383335	6	0.290905	5
**X4**	**0.282094**	**7**	**0.244807**	**9**	**0.170098**	**9**
X5	0.747219	4	0.850003	3	0.684735	3
X6	0.960079	1	0.913163	1	0.803743	1
**X7**	**0.145119**	**9**	**0.307408**	**8**	**0.260701**	**8**
**X8**	**0.034614**	**11**	**0.307410**	**7**	**0.260751**	**7**
X9	0.940824	2	0.708765	4	0.505762	4
**X10**	**0.178160**	**8**	**0.113769**	**11**	**0.074262**	**11**
X11	0.408989	5	0.419333	5	0.284607	6

Note: Bold underline means the result of PSK(sen).

**Table 5:** Redundancy analysis.

Pearson	Spearman	Kendall
**X1** ←→ **X5**	X1 ←→ X5	X1 ←→ X5
**X3** ←→ **X7**	X3 ←→ X7	X3 ←→ X7
X6 ←→ X9	**X3** ←→ **X8**	X3 ←→ X8
	**X6** ←→ **X9**	

Note: Bold means the result of PSK(red).

**Table 6:** Hyperparameter setting for single model (Y1).

Ridge	Elastic	KNN	SVM	DT	BP	RBF
alpha	l1_ratio	n_neighbors	kernel	criterion	hidden_size	hidden_size
0.2	0.3	5	Poly	gini	12	8
__	__	__	C	max_depth	learning_rate	learning_rate
__	__	__	0.7	6	0.012	0.015

**Table 7:** Hyperparameter setting for single model (Y2).

Ridge	Elastic	KNN	SVM	DT	BP	RBF
alpha	l1_ratio	n_neighbors	kernel	criterion	hidden_size	hidden_size
0.15	0.2	7	Poly	gini	9	6
__	__	__	C	max_depth	learning_rate	learning_rate
__	__	__	0.55	7	0.02	0.025

**Table 8:** Regression accuracy for single model (Y1).

Metrics\Model	Ridge	Elastic	KNN	SVM	DT	BP	RBF
MAE (Y1)	0.0581	0.0435	0.0225	0.0447	0.0173	0.0524	0.0654
MAPE (Y1)	0.1016	0.0776	0.0454	0.0638	0.0535	0.0798	0.1219
HAPE (Y1)	[2.28%, 13.54%]	[0.65%, 10.62%]	[0.59%, 6.97%]	[0.24%, 8.96%]	[0.21%, 9.61%]	[1.48%, 12.66%]	[2.76%, 15.03%]

**Table 9:** Regression accuracy for single model (Y2).

Metrics\Model	Ridge	Elastic	KNN	SVM	DT	BP	RBF
MAE (Y2)	0.0447	0.0558	0.0196	0.0382	0.0246	0.0583	0.0713
MAPE (Y2)	0.0842	0.0984	0.0524	0.0579	0.0617	0.0879	0.1037
HAPE (Y2)	[0.97%, 11.54%]	[2.25%, 13.10%]	[0.99%, 8.30%]	[1.04%, 9.76%]	[0.42%, 10.58%]	[2.58%, 11.35%]	[3.96%, 14.03%]

**Table 10:** Hyperparameter setting for MLPSA (Y1).

Layer1	Layer2	Layer3	Layer4	Layer5
Self-Attention	Dense(9,9)	Leaky_Relu	Dense(9,3)	Dense(3,1)
Optimizer	Lr	Batchsize	--	--
Adam	0.015	128	--	--

**Table 11:** Hyperparameter setting for MLPSA (Y2).

Layer1	Layer2	Layer3	Layer4	Layer5
Self-Attention	Dense(9,9)	Leaky_Relu	Dense(9,3)	Dense(3,1)
Optimizer	Lr	Batchsize	--	--
Adam	0.012	64	--	--

**Table 12:** Regression accuracy for integrated learning (Y1).

Metrics\Model	Bagging	Linear	MLP	MLPSA
MAE (Y1)	0.0185	0.0204	0.0153	0.0114
MAPE (Y1)	0.0314	0.0411	0.0337	0.0241
HAPE (Y1)	[0.33%, 6.07%]	[0.45%, 7.07%]	[0.69%, 6.19%]	[0.24%, 5.19%]

**Table 13:** Regression accuracy for integrated learning (Y2).

Metrics\Model	Bagging	Linear	MLP	MLPSA
MAE (Y2)	0.0174	0.0190	0.0135	0.0096
MAPE (Y2)	0.0278	0.0457	0.0251	0.0188
HAPE (Y2)	[0.51%, 5.91%]	[0.61%, 7.71%]	[0.57%, 8.20%]	[0.28%, 5.01%]

## Data Availability

The data is confidential due to commercial privacy.
